# COVID-19 pandemic in Economic Community of West African States (ECOWAS) region: implication for capacity strengthening at Point of Entry

**DOI:** 10.11604/pamj.2021.39.67.29089

**Published:** 2021-05-25

**Authors:** Virgil Kuassi Lokossou, Aishat Bukola Usman, Issiaka Sombie, Moussiliou Noel Paraiso, Muhammad Shakir Balogun, Chukwuma David Umeokonkwo, Josephine Gatua, John Wagai, Edgard-Marius Ouendo, Patrick Nguku

**Affiliations:** 1 Economic Community of West African States (ECOWAS) Regional Center for Disease Surveillance and Control, Abuja, Nigeria,; 2 West African Health Organisation, Bobo Dioulasso, Burkina Faso,; 3 African Field Epidemiology Network, Abuja, Nigeria,; 4 Institut Regional de Santé Publique, Ouidah, Bénin,; 5 Department of Community Medicine, Alex Ekwueme Federal University Teaching Hospital Abakaliki, Ebonyi State, Nigeria,; 6 Overseas Development Institute, London, United Kingdom,; 7 Nigeria Centre for Disease Control, Abuja, Nigeria,; 8 World Health Organization, Nairobi, Kenya

**Keywords:** Epidemic management, COVID-19 pandemic, point of entry, capacity strengthening, West Africa

## Abstract

Free movement between countries without a visa is allowed within the 15-country Economic Community of West African States (ECOWAS) region. However, little information is available across the region on the International Health Regulation (IHR 2005) capacities at points of entry (PoE) to detect and respond appropriately to public health emergencies such as Coronavirus Disease 2019 (COVID-19). ECOWAS and the member states can better tailor border health measures across the region by understanding public health strengths and priorities for improvement at PoEs. A comprehensive literature review was combined with a self-assessment of capacities at PoEs across the fifteen member states from February to July 2020. For the assessment, the member states completed an adapted World Health Organization (WHO) self-assessment checklist by classifying capacity for seven domains as fully, partially, or not implemented. The team implemented three focus group discussion (FGD) sessions and 13 key informant interviews (KII) with national-level border health stakeholders. Univariate analysis was used to summarize the assessment data and detailed content analysis was applied to evaluate FGD and KII results. Of the 15 member states, 3 (20%) are landlocked; 3 (20%) have more than one seaport. Eleven (73%) countries have 1 designated airport, 3 (20%) have two airports, and only one country (6.7%) has three airports. Two hundred and seventy-eight designated ground crossings were identified in 12 countries (80%). Strengths across the PoE were existence of decrees and ministerial acts in some ECOWAS countries and establishment of national taskforces for the COVID-19 response at PoE in ECOWAS. Major challenges were porous borders, poor intersectoral coordination, lack of harmonized traveler screening measures, shortage of staff, and inadequate financial resources. Despite all these challenges, there are opportunities such as leveraging the regional cross-border poliomyelitis coordination and control mechanism, and existence of networks of infection prevention and control specialists and field epidemiologists. However, political instabilities in some countries pose a threat to government commitments to PoE activities. The capacity to respond to public health emergencies at PoE in the ECOWAS region is still below IHR standard. Public health capacities at a majority of IHR-designated PoE in the 15-country region do not meet required core capacities standards.

## Introduction

The Economic Community of West African States (ECOWAS) was set up to foster the idea of collective self-sufficiency for each of the 15 member states. These countries have both cultural and geopolitical ties and shared common economic interest. The Atlantic Ocean forms the western and southern borders of the region. The northern border is the Sahara Desert, with the Ranishanu bend generally considered the northernmost part of the region. The eastern border lies between the Benue Trough and a line running from Mount Cameroon to Lake Chad. The vision of ECOWAS is “the creation of a borderless region where the population have access to its abundant resources and can exploit same through the creation of opportunities under a sustainable environment” [1]. ECOWAS adopted the Protocol on the Free Movement of People and Goods of 1979 to ensure free mobility of “community citizens”, i.e., citizens of ECOWAS member states, even during epidemics and pandemics [2]. This regional agreement conferred to community citizens the right to enter and reside in the territory of any member state, with a valid travel document and international health certificate. However, the same protocol also allowed member states the right to refuse admission to any community citizens who were inadmissible under the member state´s own domestic laws.

In West Africa during the Ebola virus disease epidemic of 2014 to 2016, the spread of the epidemic between countries was documented [3], resulting in the closure of borders despite the principle of free movement. With the Coronavirus Disease 2019 (COVID-19) pandemic, one of the first measures in many ECOWAS countries was the closure of air, land, and sea borders, with probable economic consequences. With the spread of COVID-19 across the ECOWAS since February 2020 [4], a growing interest in the public health response is to analyze regional and national capacities at PoEs to limit the potential spread of the infectious diseases while ensuring free movement of people and goods. Capacities at PoEs are of high interest because most of the COVID-19 index cases in West African region came into these countries through the airports [5]. An official PoE, i.e., airport, port, and ground crossing, as recognized by the decree of each country, facilitates passage for international entry or exit of travelers, baggage, cargo, containers, conveyances, goods, and postal parcels [6]. Points of entries play not only a pivotal role in enabling collaboration and coordination between countries but also in the prevention and control of the international spread of infectious diseases outbreaks. Under the International Health Regulations (IHR 2005), designated PoEs are required to establish and maintain minimum capacities including effective contingency plans, risk management, surveillance and contact tracing, communication and coordination including risk communication and community engagement, management of ill travelers, laboratory capacities, cross-border collaboration, infection prevention and control measures, environment health including sanitation and vector control, and multisectoral collaboration [7].

In May 2020, the World Health Organization (WHO) interim guidance on controlling the spread of COVID-19 at ground crossings recommended that where there are cross-border mass movements, such as displacement or migration, countries should develop and activate a COVID-19 emergency response plan. Response measures should be tailored to the risk of COVID-19 spread, based on the epidemiological situation of the country or area of origin of the travelers and coordination of opening hours and border crossing points with neighboring countries; to enable crowd management and reduce queueing [8]. A study conducted by Cliffe *et al*. reported that pandemic-related migrations are taking place within countries with some significant movements of people across borders including in Africa [8].

Risk communication and community engagement are very important in managing public health emergencies [9]. To make these strategies effective, implementers should use the most appropriate media based on preferred communication channels, such as digital media or printed materials (e.g. posters, banners, pamphlets, advisory material). Furthermore, implementers should incorporate recognition of signs and symptoms of COVID-19 and basic protective measures against the virus that causes COVID-19 into their messaging. This should be done in the appropriate languages, with an attention to literacy level, and a culturally relevant manner. The International Organization for Migration (IOM) and UNICEF have provided major support to risk communication and community engagement, especially the border communities [10, 11]. Only minimal information is available on the level of the implementation of all these public health measures at the PoEs. This paper documents the capacities at PoEs among ECOWAS member states in terms of strengths, challenges, opportunities, and provides suggestions and recommendations on how PoEs capacities can be strengthened.

## Program evaluation

### Methods

**Evaluation area:** evaluation was conducted between February and July 2020 across the 15 countries in ECOWAS region.

**Evaluation participants:** the study team invited country representatives such as IHR national focal points, heads of national public health institutes, and heads of port health services to participate.

**Data collection and analysis:** to assess capacities at PoEs in ECOWAS region, a comprehensive literature review was combined with a self-assessment of PoEs capacities by member states with an adapted checklist. Focus group discussions (FGD) and key informant interviews (KII) with relevant stakeholders were also conducted.

### Literature review

The team conducted a literature review of published (peer reviewed journal articles) and grey literature including country-level program reports, cross-border activity reports from development partners, country-specific COVID-19 response plans, National Action Plans for Health Security, standard operating procedures, regional guidelines from international organizations (World Health Organization (WHO), West African Health Organization (WAHO), ECOWAS Regional Center for Surveillance and Disease Control (RCSDC), Africa Center for Disease Control and Prevention (Africa CDC), International Civil Aviation Organization, International Organization for Migration (IOM), Mano River Union, and others) to explore the progress on, challenges to, opportunities for, and threats to managing public health events at PoEs. The team specifically sought to identify strengths and gaps in strengthening capacities at PoEs. The team searched in electronic databases, including Google Scholars, PubMed, African Journals online, Web Science, and ECOWAS web pages, for articles and published activities on PoEs in West Africa to highlight progress made and existing gaps during 2015 to 2019. We focused on this period because the most important developments in strengthening PoEs capacities started during and after the 2014 to 2016 Ebola virus disease epidemic in West Africa. Reports of the Joint External Evaluation (JEE) organized by WHO and partners for PoEs across the 15 West African countries were also reviewed [12]. The two indicators for PoE in the JEE tool were routine capacity established and effective public health response at PoE. These indicators were graded on a scale of 1-5: 1 = nonexistent capacity; 2 = limited capacity; 3 = capacity developed; 4 = demonstrated ability; 5 = sustainable capacity. Data were extracted into Microsoft Excel. We summarized the extracted data and reported on the strengths, challenges, opportunities, and threats.

### Self-assessment of capacities at points of entries

In February 2020, a self-assessment checklist was sent via email to IHR national focal points and relevant authorities in charge of PoE in all ECOWAS Member States to help them summarize their national context about PoEs. This checklist was adapted from the IHR assessment tool for capacity requirement at designated ports, seaports and ground crossings [13] and was used to assess PoEs in seven domains: legal enforcement and planning, surveillance (early detection), surveillance (interview and management of ill travelers suspected of COVID-19), acute emergency response during mass movement across the border, supplies for infection prevention and control, risk communication and community engagement, and cross-border communications. Each of these indicators was graded as fully implemented (Y), partially implemented (P) or not implemented (N). The responses were collated and entered into Microsoft Excel to generate frequencies and percentages which are summarized in tables.

### Consultation phase

**Figure 1:**
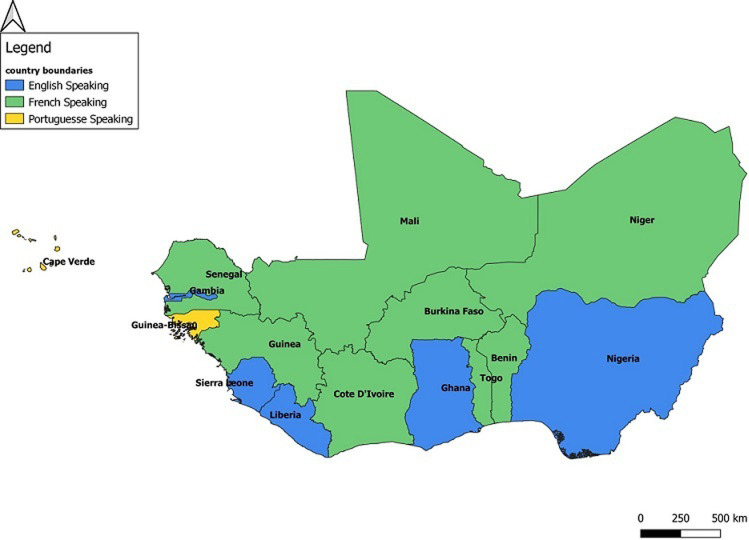
categorization of 15 Economic Community of West African States (ECOWAS) member states by language for focus group discussions on border health capacities and during the COVID-19 pandemic, February-July 2020

We conducted 14 in-depth, 40-60-minutes interviews (KII) with representatives from 13 countries: Burkina Faso, Cabo Verde, Côte d´Ivoire, The Gambia, Ghana, Guinea, Liberia, Mali, Niger, Nigeria, Senegal, Sierra Leone, and Togo. Country representatives were IHR national focal points, heads of national public health institutes, and heads of port health services. In some countries, the one person may hold more than one of these roles. Interviews were designed to gather information on the number and types of IHR-designated PoE, availability and implementation of public health emergency response plans at PoEs, measures carried out at the PoEs before the declaration of the COVID-19 pandemic ,capacity building of PoEs staffs in terms of training, and provision of essential health interventions (for example, surveillance, case management, infection prevention and control, risk communication), and actions or measures PoEs took during the pandemic response and associated challenges. The discussions were recorded and transcribed verbatim. Three virtual FGD were held between countries by language groups (English, French, and Portuguese) as shown in Figure 1. FGD participants included national authorities responsible for implementing IHR; heads of national public health institutes; public health professionals involved in disease surveillance, risk communication, emergency preparedness and response, animal health, and environmental health at PoEs and in nearby communities; representatives of non-governmental organizations and partners such as WHO-Africa Regional Office, IOM, ECOWAS Regional Center for Surveillance and Disease Control, ICAO, WAHO, West African Economic and Monetary Union (WAEMU), Mano River Union, African Field Epidemiology Network (AFENET), African Development Bank, World Bank, Abidjan-Lagos Corridor Organisation (ALCO), Pro-health International, United States Centers for Disease Control and Prevention (US CDC), the German Technical Cooperation (GIZ), and other partners at ground crossings. The virtual group meetings were held to better understand how the region´s PoEs are organized and to explore similarities and differences in PoEs management. These discussions were recorded and subsequently translated to English for French- and Portuguese-speaking countries. All recordings as well as notes taken were transcribed verbatim, analyzed with NVIVO version 8 [14] and summarized to categorize the strengths, opportunities, and challenges of the national and regional initiatives, planned strategies, and ongoing preparedness activities for COVID-19 pandemic within the ECOWAS region. Results were compared with findings of the in-depth interviews.

### Ethical considerations

The purpose of the evaluation was explained to lead representatives of member states before the commencement of data collection. Informed consent was obtained from the participants, and participants understood that they had the right to withdraw their consent to participate at any stage of the assessment without any consequences to themselves, their respective agencies, or countries. Anonymity was not guaranteed; however, confidentiality and privacy of data were maintained. Most data were analyzed at the aggregate level by country, with a few exceptions where expressed permission was obtained to include quotes of some interviewees.

## Results

### Distribution of PoEs in ECOWAS

Of the 15 member states, 3 (20%) are landlocked while 3 (20%) have more than one designated seaport (Table 1). Eleven countries have only one designated airport (73%), three countries have 2 designated airports, and only one country (6.7%) has three designated airports. Two hundred and seventy-eight designated ground crossings were identified in 12 countries (80%) while 3 countries did not have any. Official PoEs as recognized by the decree of each country were present in 12 (80%) with three countries having more than hundred. In most of the countries, there are uncountable unofficial /illegal ground crossing points as revealed during an in-depth interview, “we can´t count the number of ground crossings. This is the big problem we are dealing with. We have several of team without port health staff; that´s a very big gap” -Key Informant 2. The large number of ground crossing raised the challenge of lack of skilled human resources to cover all the PoEs. Most official PoEs only have a minimum capacity in place, as supported by a FGD finding, “These ground crossings are many, especially the ones that leads to markets, and there is no building or constructions there for staff to stay even though we have military in some of the places” - FGD Participant 5. The ECOWAS region is faced with the issue of porous borders (entry of people through unofficial border crossings following border closures) as highlighted by FGD -1 Participant 4: “Borders are closed but people still continue entering and luggage circulation. A tool has been developed to collect identity, symptoms and signs to be followed up in the county.”

**Table 1:** distribution of Points of Entry (PoE) in the ECOWAS Region

Countries	Designated Seaports	Designated Airports	Designated Ground PoE	Official Ground PoE
Benin	1	1	11	NA
Burkina Faso	NA	2	12	32
Carbo Verde	2	2	NA	NA
Cote d´ivoire	1	1	5	43
Gambia	1	1	16	NA
Ghana	2	1	14	41
Guinea	1	1	11	30
Guinea Bissau	3	1	7	24
Liberia	1	2	45	131
Mali	NA	1	54	9
Niger	NA	1	54	NA
Nigeria	1	3	NA	51
Sierra Leone	1	1	4	151
Senegal	1	1	NA	75
Togo	1	1	5	15
**Total**	15	20	278	600

### Capacities at PoEs according to JEE in ECOWAS region

**Figure 2:**
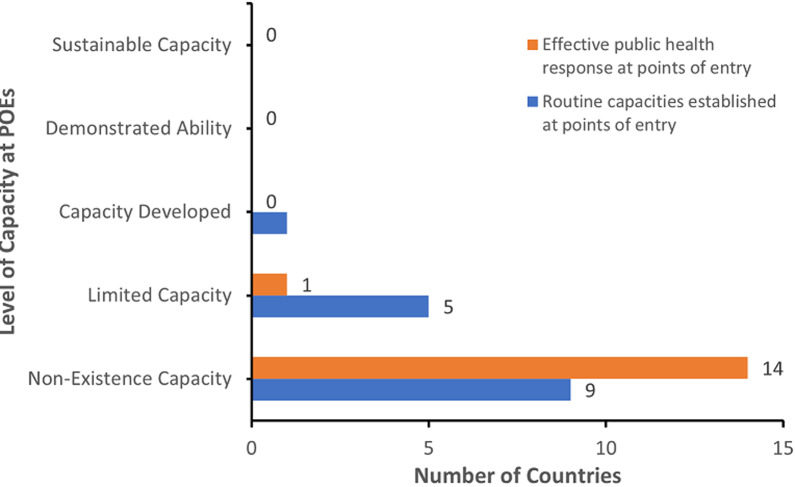
summary of joint external evaluation results for Point of Entry capacities in Economic Community of West African States (ECOWAS), 2016-2017

The analysis of JEE reports in the ECOWAS region showed that 93% and 60% of the member states did not have routine capacities established or effective public health response at points of entry, respectively (Figure 2). This finding was confirmed during interactions with key informants who stated that: “Most of the PoEs never had functional staff and structures in place before JEE. Most of the discussions started during and after the Ebola outbreak; that was when the role of PoEs was being appreciated with some investment on PoEs” - Key Informant 5.

This analysis also revealed that several initiatives for strengthening capacities at PoEs really started in West Africa after the JEE. This was corroborated by Key Informant 3: “After JEE, we have developed contingency plan and clear standard operating procedures (SOPs). Simulation exercises (were) done even though (there was) no systematic mechanism for this to be done bi-annually with airports, seaport and land crossings”. During KII participants revealed that some of the PoEs were lacking resources such as personnel protective equipment (PPE), guidelines and SOPs and capacities for delivering emergency first aid to ill travelers. Systems to refer ill travelers for health care were not in place in most of the countries as revealed by Key Informant 3, “Another key indicator for JEE is the first aid emergency at the PoE. I don´t think we have adequately positioned this, the fact that we have deployed personal protective equipment, we have paramedical staffs that needs to be trained in providing first aid services except ambulance staffs”.

### Assessment of capacity requirement at PoE in the ECOWAS Region

Before the COVID-19 pandemic, 2 countries (16.7%) had fully implemented legal enforcement and planning, but this increased to 5 (33.3%) during the pandemic while 9 countries (60%) maintained the status quo of partial implementation (Table 2). This finding was corroborated by an in-depth interview “At the ground crossings efforts are being made to make contingency plans available, but at draft stage. There are SOPs for airport, which was easy to adopt, now because of COVID-19 supplies for IPC (infection prevention and control) and staffs are being trained. But at seaport (there is) no contingency plan and SOPs but there (is) a bit of training here and there and supplies” - Key Informant 5.

**Table 2:** assessment of capacity requirement at Points of Entry (PoE) in the Economic Community of West African States (ECOWAS) region before and during the COVID-19 pandemic, February-July 2020

Indicators	Before COVID-19 (< December 2019)	During COVID-19 (≥ December 2019)
**Legal enforcement and planning**	**Frequency (%)**	**Frequency (%)**
Fully implemented	2 (13.3)	5 (33.3)
Partially implemented	9 (60.0)	9 (60.0)
Not implemented	4 (26.7)	1(6.7)
Surveillance: Early Detection		
Fully implemented	7(46.7)	14(93.3)
Partially implemented	8(53.3)	1(6.7)
Surveillance: Interview and management of ill travelers suspected of COVID-19		
Fully implemented	9(60.0)	14(93.3)
Partially implemented	6(40.0)	1(6.7)
Acute emergency response during mass movement across the border		
Fully Implemented	8(53.3)	11 (73.3)
Partially Implemented	7(46.7)	4(26.7)
Supplies for infection prevention and control		
Fully implemented	6(40.0)	10(66.7)
Partially implemented	7(46.7)	5 (33.3)
Not implemented	2(13.3)	0(0.0)
Risk communication and community engagement		
Fully implemented	5(33.3)	10(66.7)
Partially implemented	9(60.0)	5 (33.3)
Not implemented	1(6.7)	0(0.0)
Cross-border collaboration		
Fully implemented	1(6.7)	2(13.3)
Partially implemented	1(6.7)	3(20.0)
Not implemented	13(86.7)	10(66.7)

All the ECOWAS countries had surveillance systems for early detection in place at PoEs before the COVID-19 pandemic, but most (53.3%) were partially implemented. There were significant efforts during the pandemic to achieve the stipulated standard of the IHR (2005) by having 14 (93.3%) meeting up to standard and interview and management of ill travelers suspected of COVID-19 was 40% fully implemented but during the pandemic this rose to 93.3% (Table 2). The qualitative findings showed that most of the efforts were at the airports leaving ground crossings and seaports insufficiently supported. “In the airport, there are holding facilities for suspected cases until they are investigated except if the passenger present with respiratory symptoms, then ambulance can take the person to designated treatment center. We have not been able to successfully establish that at the land crossings, only few facilities (have been) sustained since Ebola outbreak. More than 95% are temporary structure to conduct primary screening” -Key Informant 4.

Eight (53.3%) of the ECOWAS countries had measures in place before the COVID-19 pandemic for acute emergency response during mass movement across the border, but during the pandemic 11 (73.3%) countries implemented the needed measures fully. Less than half (40%) of the member states fully implemented infection prevention and control, but this increased significantly to 66.7% during the period that COVID-19 was being identified in these countries (Table 2). There were PPE shortages, health workers infections and improper management of crowds as revealed by the following key informants and FGD participants: “emergency mass movement has been one of the issues we have addressed. We developed some guidelines for huge populations assembling for services like Bank. However, what has been challenging to us is the supervisory aspect of this due to inadequate resources” - Key informant 4.

“We still don´t have adequate PPEs. At times we run out of gloves and the tap at washing stations usually run out of water. If water is not running, we make use of bucket water” - FGD 1 Participant 3.

“For the last one week, 70% of the confirmed cases have been health care workers. It´s extremely worrisome for all of us. Training on IPC at health facility and PoE is extremely critical at this time but how do we ensure compliance? The fact that we are having increase in health care worker infection suggest something is wrong. The initial assessment that was done shows frequent stock out of PPEs and issue of compliance” -Key Informant 3.

This evaluation showed risk communication and community engagement improved to 66.7% during COVID-19 as compared with 33.3% before the pandemic. Qualitative findings revealed that there were community engagement and risk communication activities on COVID-19 within the region. “There are banners and posters at airport which shows sign and symptoms of COVID-19 and what you must do if you are suspecting COVID with numbers to call. Also, information leaflets available for passengers to read and follow preventive measures” -Key informant 6.

“At ground crossings, risk communication pillar and IOM are supporting; focusing on border communities and illegal land crossings to engage them on using megaphone” -FGD 3 participant 1. Cross-border cooperation is defined as a set of arrangements and actions jointly carried out by various stakeholders on both sides of an international border, with the aim of improving the capacity to prevent, detect, and respond to public health risk events across borders. Cross-border cooperation and interventions to be carried out at PoEs, include: surveillance of diseases and other events that may become public health emergencies; mechanisms for facilitating and providing quality care to cross-border populations; harmonization of surveillance methods, approaches and tools; pooling of health resources on both sides of the border; mechanisms for information sharing; and how cross-border cooperation builds and enhances existing capacities in a cross-border framework. With regards to cross-border collaboration, only 6.7% of the member states have fully implemented cross-border collaboration and during the pandemic the percentage collaboration is 13.3% with neighboring countries (Table 2). Discussions with key informants revealed that some level of collaboration does exist among neighbouring countries such as the friendship and cooperation treaty between Burkina Faso and Côte d´Ivoire; the agreement (albeit unsigned) on cross-border cooperation between Guinea and Sierra Leone; ongoing cross-border cooperation between Guinea, Sierra Leone, and Côte d'Ivoire; and cross-border cooperation mechanism between Mali and its seven neighbouring countries (Algeria, Burkina Faso, Côte d'Ivoire, Guinea, Mauritania, Niger and Libya). “Yes, we have border collaboration with neighboring countries, we have MOU (memoranda of understanding) signed with them. They send in their weekly surveillance report to us and (we) send ours too but with border closure now that has not happened” - Key informant 6.

### Strength, challenges, opportunities and threats at the PoE in the ECOWAS Region

Table 3, Table 3 (suite), Table 3 (suite 1) show the strengths, challenges, opportunities, and threats analysis of IHR core capacities such as PoE organization, coordination, legal enforcement and planning, surveillance, early detection and management of cases, infection prevention and control, risk communication and community engagement, infrastructure, capacity building, workforce, management and transportation of ill travelers, and community engagement. The key strengths were existence of decrees and ministerial acts in some ECOWAS countries, availability of international guidance documents such as the IHR (2005), establishment of national taskforces focusing on the COVID-19 response at PoEs in the ECOWAS region, cross-border cooperation mechanism between Mali and its seven neighboring countries, and existence of the networks of national public health institutes and port health services. The keys challenges were porous borders, strategies to detect ill travelers at the PoE and monitor travelers for illness after arrival, community stakeholder engagement, poor intersectoral coordination, lack of harmonized traveler screening measures with other countries, shortage of staff to cater for all PoEs, insufficient material and financial resources for the implementation of surveillance activities at PoE, and lack of a framework/platform for exchanges of information between PoE stakeholders. Despite all these challenges there are opportunities within the PoE such as leveraging on the regional cross-border poliomyelitis and Lassa fever coordination and control mechanism willingness of technical and financial partners to support PoE activities (WHO, AFENET, Africa CDC, IOM, ALCOL, Pro Health International, US CDC), existence of network of infection prevention and control specialists, availability of training centers and a network of field epidemiologists. However, political instabilities in some countries pose a threat to government commitments to PoE activities.

**Table 3:** strengths, challenges, opportunities and threats at PoEs in the Economic Community of West African States (ECOWAS) region, February - July, 2020

	Strengths	Challenges	Opportunities	Threats
**Organization of PoEs**	Existence of decrees and ministerial acts in some ECOWAS countries Designation of official POEs according to the provisions of IHR (2005). High turnover of staff managing PoEs in ECOWAS countries	Porous borders (entry of people through unofficial border crossings following border closures), ill traveller detection and traveller monitoring strategy, community stakeholder engagement. High number of non-designated POEs.	Advocacy to high- level authorities in the region	Political instabilities
**Coordination, Legal Enforcement and Planning**	Availability of international guidances such as IHR (2005) Availability of political commitment for POEs and cross-border activities such as Protocol of creation of WAHO, Guidelines for harmonizing the movement of persons in ECOWAS region, Existing collaboration mechanisms in West Africa (UEMOA, Mano River Union Keen interest of Member States in adapting plans and SOPs for facilitating information sharing at PoEs. Establishment of National taskforces focussing on the COVID-19 response at POEs in ECOWAS region. Existence of the networks of national public health institutes and port health services	Poor coordination of intersectoral collaboration. Inadequate or lack of cross-border cooperation. Lack of response plans for public health emergencies specific to each designated PoEas recommended in the IHR (2005). Lack of harmonized traveller screening measures with other countries. Lack of a framework/platform for exchanges between POE stakeholders. Need for holding regular simulation exercises. Lack of standard operating procedures in some POEs. Lack of coordination of PoEs s activities at national level across sectors	Willingness of technical and financial partners to support points of entries activities (WHO, AFENET, Africa CDC IOM, ALCOL, Pro Health International)	Political instabilities in some countries thus affecting government commitments into PoE activities.

**Table 3(suite):** strengths, challenges, opportunities and threats at PoEs in the Economic Community of West African States (ECOWAS) region, February - July, 2020

	Strengths	Challenges	Opportunities	Threats
Surveillance, Early detection, and Management of cases		Insufficient material and financial resources for the implementation of surveillance activities at PoE Lack of capacity to manage travellers´ information (Surveillance) Lack of guidance for the management of quarantine during lockdowns. Lack of training and deployment of Rapid Response Teams in PoEs for strengthening the detection, isolation, and quarantine of travellers	High turn-over of technical experts at the country level	
Infection Prevention and Control		Reluctancy of travellers to perform handwashing or hand-disinfection with alcohol-based hand sanitizer. Insufficient personal protective equipment at POEs Lack of running water in some POEs for handwashing. Lack of waste management capacities at PoEs	Existence of network of infection prevention and control specialists	
Risk Communication and Community Engagement		Lack of engagement/buy-in of cross-border communities. Passenger impatience during awareness sessions on COVID-19		
Infrastructure		Insufficient and inadequate isolation rooms/holding areas and case management at PoE. Lack of workspace, screening rooms and isolation centres at some points of entry. Non-existent of quarantine stations for imported animals owing to the potential public health risks, with the use of a multi-sectoral approach (One Health Approaches). Irregular electricity supplies in some PoEs.	Availability of technical and financial partners	

**Table 3(suite 1):** strengths, challenges, opportunities and threats at PoEs in the Economic Community of West African States (ECOWAS) region, February - July, 2020

	Strengths	Challenges	Opportunities	Threats
Workforce at PoEs		Shortage of staff to cater for all PoEs. Not all border crossings are currently equipped with port health personnel, thus posing a challenge to the surveillance system. Shortage of financial resources to implement activities at PoEs. Shortage of requisite medical supplies, materials, and equipment, including reagents (with the onset of the pandemic). Delays in mobilizing resources. Non-availability of a control mechanism at the onset of the pandemic. Low motivation among country stakeholders. Inadequate training and assignment of qualified personnel for inspection of conveyances at the official PoE.	Availability of training centers and network of field epidemiologists	
Traveller management and transportation		Lack of a carrier tracking system (carrier manifest) in West Africa raising contact tracing issues. Low passenger registration capacity (completion of check-in forms) due to massive population movements before border closures. Difficulty in identifying passengers who change seats while on board, which poses a risk for COVID-19 transmission. Flight manifests are sometimes inaccurate. Lack of cooperation from passengers, thus complicating the screening process and transfer to designated quarantine facilities. Non-compliance with procedures by passengers and drivers. Challenges in implementing social distancing measures		
Cross- border Collaboration	Cross-border cooperation mechanism between Mali and its seven neighbouring countries (Algeria, Burkina Faso, Côte d'Ivoire, Guinea, Mauritania, Niger, and Libya).	Lack of engagement/buy-in of cross-border communities. Passenger impatience during awareness sessions	Leveraging the regional cross-border poliomyelitis coordination and control mechanism and Lassa fever	

## Discussion

In the ECOWAS region, the establishment of PoEs is decided by a government order signed by either the President´s or Prime Minister´s Office (a Decree) or the Ministry of Public Health (an Order). This decree or order lays down the legal rules and regulations for the operation and management of health control points. All the countries have an established number of official border crossings and have mutually agreed on the most important ones (PoE designated to meet core capacities specified in the IHR). The number of official or designated PoE varies markedly from one country to another. Findings from this evaluation showed that there are numerous unofficial border crossings in the West African region which have posed a lot of challenges in the fight against COVID-19 pandemic and other human and animal diseases. Studies have shown that one of the major consequences of porous borders in West Africa today is the rise of trans-border crimes and security threats such as human trafficking [15]. The problem lies in the complexity of the organizations of these borders and their activities, the global penetration, and the threat they pose to democracy and legitimate economic development.

The JEE is part of the WHO´s new process to help countries assess their ability to prevent, detect and respond to public health threats such as infectious disease outbreaks, as specified by IHR. The review of JEE reports for the region revealed important gaps in routine capacity and effective public health response at point of entry. This finding is in line with the conclusion of a study that poor performance on PoE indicators suggest that countries are not only ill-prepared for cross-border outbreaks but are struggling to provide key public health services that are critical to keeping their populations healthy and safe [16]. Another study conducted in the African region reported major gaps and inadequate and untimely resources for PoEs [17]. Without these core capacities in place at PoEs, future outbreaks may become larger-scale pandemics than the COVID-19 the world is experiencing now. One fact that is clear is further investments are needed that specifically target preparedness and core public health functions represented by the JEE for PoEs. During public health emergencies of international concern, the WHO recommends the review of national and local legislative requirements for implementation of necessary health measures at various points of entry including development, improvement, and implementation of public health emergency contingency plans in line with IHR (2005) Annex 1 on PoE capacities requirements (WHO, 2012). Most member states do have contingency plans and SOPs in draft form (not finalized). A study conducted among mobile populations along porous borders of Nigeria, Togo and Benin which examined the development of PoE-specific public health emergency response plans and SOPs found that, at many PoEs, individual agencies often know appropriate procedures to take during a public health event, yet the procedures are not documented or shared [18]. In the absence of an agreed-upon plan, stakeholders risk gaps or redundancies in communication, surveillance, and response efforts, consequently increasing the risk of an uncoordinated and delayed response.

The WHO has recommended training of health care workers including border health staff on infection prevention and control requirements for staff and travelers, how to screen travelers, use of noncontact infrared thermometers, use of PPE during the screening process, and implementation of paper-based and/or electronic systems for storing passengers´ information [19]. A global analysis on infection prevention and control strategies found that more than 22,000 frontline workers have been infected across 55 countries, the infection prevention and control guidelines fail to cover all transmission modes, and the recommendations also conflict with each other. It then concluded that infection prevention and control strategies should consider all the possible routes of transmission and should target all patient care activities involving risk of person-to-person transmission. There is a need to have a standardized protocol or guidelines across the region that guides the usage of PPE and other infection prevention and control measures [20]. The current COVID-19 pandemic has spread across borders through travelers, and various means of transportation, which has prompted demands for the detection and management of suspected cases at PoEs, including ports, airports, and ground crossings, and on-board conveyances. Our findings showed that most of our member states fully implemented the WHO recommendations for interviewing and management of ill travelers. This may be because the ECOWAS region was affected late, with its first case in late February 2020 in Nigeria, whereas the first cases in Africa were reported to WHO early in January 2020, giving the region some level of opportunity to prepare ahead. According to an assessment conducted by IOM on PoEs in Ghana, 71% of PoEs have PPE available for use during the screening of passengers while only 22% had installed the necessary infrastructure to support crowd control and ensure safety of passengers [21]. Similarly, this evaluation found that most efforts are concentrated in the airports while other PoEs hardly have structures in place.

### Way forward

Based on our findings and to enhance capacities at PoEs in ECOWAS region according to IHR (2005) requirements for COVID-19 pandemic response, we would like to propose following suggestions to the policy makers:

**Governance and leadership:** development and implementation of an ECOWAS regional strategy for multisectoral and multi-country collaboration, partnerships, and networks.

**Funding:** setting up a sustainable (internal and external) and multisectoral funding mechanisms for COVID-19 pandemic preparedness and response activities and other public health emergencies at PoEs.

**Human resource and capacity building:** creating and operating a regional platform or network of PoE workers in the ECOWAS region and development of capacity building plan and harmonized training curriculum for PoE workers.

**Operations and Service Delivery:** strengthening the organization of PoEs (air, sea, and land); enhance necessary infrastructure (workspace for teams) and provide work equipment including adequate supplies of personal protective equipment; strengthen the public health intervention package (epidemiological surveillance, laboratory, case management, infection prevention and control; risk communication and community engagement) for emergency preparedness. Health Information Systems: promote a digital platform for real-time or near real-time information sharing within the ECOWAS region. Cross border coordination: Support the implementation of ECOWAS guidelines on the COVID-pandemic and related recovery actions for the harmonization and facilitation of cross-border trade and transport and mitigation of health risks in the ECOWAS region, validated by the 58^th^ ordinary session of the authority of heads of states and governments on 23^rd^ January 2021.

**Limitations of this evaluation:** the opinions expressed during FGD and KII and the responses from the self-assessment checklist by the representatives of the member countries may not completely reflect the reality in each country. Also, this study was conducted early in the pandemic and may not reflect improvements made by each country since then.

## Conclusion

West Africa is facing the COVID-pandemic and other outbreaks of infectious diseases. Responses to previous public health emergencies that involved cross-border spread of infectious diseases emphasize the important role of preparedness at PoE. The proposed strategies will enable the ECOWAS countries to respond more effectively to future infectious disease emergencies and help achieve the ECOWAS vision including free movement in the region.
